# The P300 Event-Related Potential Component and Cognitive Impairment in Epilepsy: A Systematic Review and Meta-analysis

**DOI:** 10.3389/fneur.2019.00943

**Published:** 2019-08-30

**Authors:** Rui Zhong, Mengmeng Li, Qingling Chen, Jing Li, Guangjian Li, Weihong Lin

**Affiliations:** ^1^Department of Neurology, The First Hospital of Jilin University, Changchun, China; ^2^Department of Hepatology, The First Hospital of Jilin University, Changchun, China

**Keywords:** P300—event related potential, cognitive impairment (CI), epilepsy, meta-analysis, P300 latency, P300 amplitude

## Abstract

**Background:** Epilepsy is one of the most prevalent chronic brain diseases worldwide and is often accompanied by cognitive impairment. Event-related potentials (ERPs) are an objectively non-invasive approach for studying information processing and cognitive functions in the brain. The P300 is an important and extensively explored late component of ERPs that has been widely applied to assess cognitive function in epilepsy in previous studies. However, consistent conclusions have not yet been reached for various reasons.

**Objective:** We conducted a comprehensive systematic review and meta-analysis of P300-related studies to assess the latency and amplitude of the P300 in epileptic patients.

**Methods:** PubMed, EMBASE, and Cochrane Library databases were systematically searched for eligible studies. The standard mean difference (SMD) and the 95% confidence interval (CI) were calculated as the effect size of the P300 component.

**Results:** The main results of the present meta-analysis indicated that epileptic patients have a longer P300 latency and a lower P300 amplitude than controls. Subgroup analysis based on age group demonstrated that these differences can be observed in both children and adult patients compared with healthy controls. In addition, the P300 latency was longer in patients with the five main types of epileptic seizures than in controls.

**Conclusion:** This study revealed that epileptic patients have abnormalities in the P300 component, which may reflect deficits in cognitive function. Thus, the P300 may be a potential objective approach for evaluating cognitive function in epileptic patients.

## Introduction

Epilepsy is one of the most prevalent chronic brain diseases worldwide and is characterized by abnormal, excessive, and synchronous neuronal activity (1). It is estimated that more than 60 million individuals are affected by epilepsy worldwide, making up 0.6% of the global economic cost of the disease, as this disease severely reduces quality of life and increases the risk of mortality (2). Cognitive dysfunction occurs in approximately 70–80% of epileptic individuals (3). In addition, recent research has demonstrated that 50% of newly diagnosed and untreated epileptic patients had cognitive impairments, and cognitive impairments were present in 64.5% of epileptic children with abnormal brain imaging (4, 5). The significant association between the frequency of epileptic seizures and cognitive impairment was identified by various studies (4, 6). In addition, the cognitive status of epileptic individuals is also affected by other factors, such as age of onset, type of epilepsy, duration, use of antiepileptic drugs (AEDs) and so on (7–9).

Event-related potentials (ERP) are an objectively non-invasive approach for studying information processing and cognitive brain functions, such as attention, learning, memory, and decision-making, that are characterized by their positive or negative polarity, latency, and high temporal resolution (10, 11). Sutton et al. first reported a evoked potential component that reached its peak amplitude at approximately 300 ms and found a significant association between this ERP component and cognitive function (12). Subsequently, it has been identified by many studies that cognitive impairment in various brain diseases, such as Alzheimer's disease, Parkinson's disease, epilepsy, and stroke, can be objectively assessed by ERPs (13–17). Mild cognitive impairment (MCI) refers to a prodromal stage of Alzheimer's disease (AD) that exceeds typical “age-related” reduction in cognition but does not fulfill the criteria for AD (18). A recent meta-analysis indicated the effectiveness of ERPs in the early diagnosis of MCI.

The P300 is an important and extensively explored late component of ERPs that is widely applied to assess cognitive function in humans (12). The amplitude and latency of the P300 component provides information about cognitive processes in the brain, such as memory, attention, concentration and speed of mental processing (13). Many previous studies have focused on the application of the P300 component in the assessment of cognitive impairment in epileptic patients; however, consistent conclusions were not reached for various reasons. In addition, some studies did not have sufficient statistical power due to a limited sample size. Considering these factors, we conducted a comprehensive systematic review and meta-analysis of P300-related studies to assess the latency and amplitude of the P300 in epileptic patients. This was the first meta-analysis to summarize all of the epilepsy-related P300 studies. We hypothesized that the P300 may be sensitive to detecting cognitive impairment in epileptic patients.

## Methods

### Search Strategy

PubMed, EMBASE, and Cochrane Library databases were systematically searched for eligible studies by two independent reviewers (Zhong and Chen) from inception to 1st Feb 2019. The search terms were used as follows: (“event-related potential” OR “ERP” OR “P300” OR “P3”) AND (“epilepsy” OR “seizure”). The publication language was restricted to English. The reference lists of eligible studies were also reviewed to identify additional articles. Two authors independently reviewed the title and abstract of each paper. After excluding obviously irrelevant articles, the authors examined the full text of the selected articles and decided the exact list of literature to be included in the present meta-analysis. The authors resolved disagreements by discussion.

### Selection Criteria

An eligible study should fulfill the following criteria: (1) The study design was a case-control study, cross-section study or cohort study; (2) the trial included epileptic patients and healthy controls; (3) the amplitude and the latency of the P300 component were measured by the ERP technique and were pooled; and (4) the amplitude and latency of the P300 component were compared between epileptic patients and controls. Duplicates were removed. Reviews, case reports, meta-analyses and conference abstracts were also excluded. Two reviewers (Zhong and Chen) independently carried out study selection.

### Data Extraction and Quality Assessment

The following data were extracted by two independent reviewers: first author, publication year, population based on age group (children or adults), sample size, electrode location and stimuli (Hz). We also extracted the amplitude and latency of the P300 component in epileptic patients and controls. Two reviewers (Zhong and Chen) independently extracted data from each eligible study.

The Newcastle-Ottawa Scale (NOS) was used to assess the quality of each eligible study. Studies with a score of six or higher were categorized as being high quality studies. Studies that scored between zero and five were regarded as being low quality. Two reviewers independently evaluated the quality of the included studies.

### Statistical Analysis

The standard mean difference (SMD) and the 95% confidence interval (CI) were calculated as the effect size of the P300 component. We used Cochran's Q statistic and the *I*^2^ statistic to assess heterogeneity across studies. *P* < 0.1 or *I*^2^ > 50% indicated significant heterogeneity. We used the random effects model if heterogeneity was present in the data; otherwise, we chose the fixed effects model. Subgroup analysis based on age-group population and type of epileptic seizure was carried out to explore potential sources of heterogeneity. Sensitivity analysis was also conducted to test the stability of the results by removing one study at a time. Publication bias was described using funnel plots. The cognitive status of epileptic patients was affected by various factors, such as the type of epilepsy, duration, and use of AEDs. Thus, the differences in the amplitude and latency of the P300 component across different types of epileptic cases were also calculated by meta-analysis. The Review Manager Software Package (RevMan 5.3) was used to conduct all statistical analyses.

## Results

### Search Results and Study Characteristics

**Figure 1:**
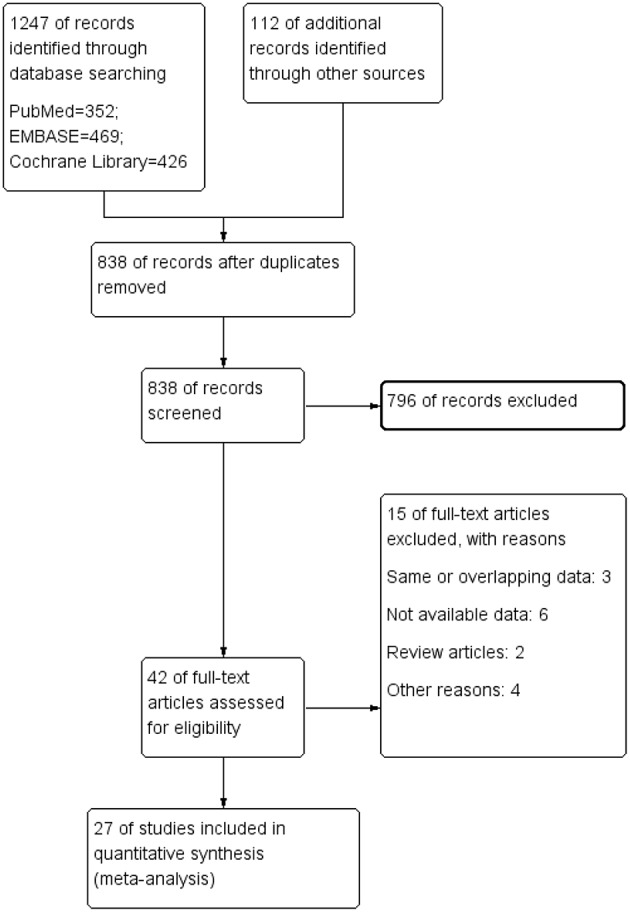
The process of study identification.

A total of 1,359 potentially eligible references were initially identified based on our search strategy. A total of 521 articles were removed due to being duplicates, and 838 articles remained. Then, we excluded 796 articles after screening the title and abstract. A total of 42 articles remained for full text reading. Eventually, 27 eligible studies (13, 19–44) containing 1,513 epileptic patients and 1,124 healthy controls were included in our meta-analysis. Figure 1 shows the study selection process.

A total of 27 eligible studies were included in the present meta-analysis. Of these studies, 10 included children and adolescent patients with epilepsy, and 17 studies were based on adult epileptic cases. The locations of the electrodes and the stimuli (Hz) used in the tasks were substantially variable across studies. The Cz electrode and 1,000/2,000 Hz stimuli were most frequently used in these studies. Most included studies had a quality score over 6, according to the Newcastle-Ottawa Scale, indicating good quality in general. The main basic characteristics and quality assessments of the included studies are summarized in Table 1.

**Table 1:** The main basic characteristics and quality assessment of the included studies.

**References**	**Age group**	**Number of**		**Site**	**Frequent/rare stimuli(HZ)**	**Quality**
		**EG**	**CG**			
Elsawy et al. (19)	Adults	30	30	Cz	2,000/8,000	6
Fosi et al. (20)	Children	22	22	Cz, Fz, T3, T4	1,000/2,000	8
Takhirovna et al. (21)	Adults	75	30	C3, C4	1,000/2,000	7
Casali et al. (13)	Children	20	16	Fz	1,000/2000	7
Boscariol et al. (22)	Children	19	16	Fz	1,000/2,000	7
Artemiadis et al. (23)	Adults	16	43	CPz, AFz	1,000/2,000	5
Watanabe et al. (24)	Adults	14	14	Cz, Fz, Pz	1,000/1,050	7
Tumay et al. (25)	Adults	53	20	Cz, Pz	1,000/2,000	6
Rocha et al. (26)	Adults	12	12	C2, C4	1,000/1,500	6
Ivetic et al. (27)	Adults	35	20	Cz, Fz	1,000/2000	5
Sun et al. (28)	Adults	30	15	C3, C4, P3, P4	NR	7
Duman et al. (29)	Children	21	21	Cz, Fz	1,000/2,000	7
Chayasirisobhon et al. (30)	Adults	30	30	Cz, Fz, Pz	1,000/2,000	6
Soyuer et al. (31)	Adults	73	31	Cz, Fz	1,000/2,000	7
Gokcay et al. (32)	Children	30	25	Cz, Fz	1,000/8,000	7
Celebisoy et al. (33)	Children	120	25	Cz	1,000/8,000	6
Turkdogan et al. (34)	Children	50	21	Cz, Fz, Pz	1,000/8,000	6
Chen et al. (35)	Adults	27	60	P3, P4	125/750	6
Caravaglios et al. (36)	Adults	108	32	Cz, Fz, Pz	1,000/2,000	6
Soysal et al. (37)	Adults	116	15	Cz, Fz	1,000/8,000	6
Kubota et al. (38)	Adults	120	78	Cz, Fz, Pz	1,000/2,000	7
Wu et al. (39)	Adults	50	38	Cz, Fz,C3, Oz	1,000/2,000	6
Shimono et al. (40)	Adults	12	9	Cz, Fz, Pz	1,000/2,000	6
Naganuma et al. (41)	Children	72	67	Pz	1,000/2,000	6
Konishi et al. (42)	Children	129	53	Pz	1,000/2,000	6
Naganuma et al. (43)	Children	23	54	Pz	1,000/2,000	6
Triantafyllou et al. (44)	Adults	68	30	Cz, Fz, Pz	1,000/2,000	6

*EG, epilepsy group; CG, control group; NR, not reported; Site, location of electrodes*.

### Meta-analysis Results for the Latency and Amplitude of the P300 in Epilepsy

**Figure 2:**
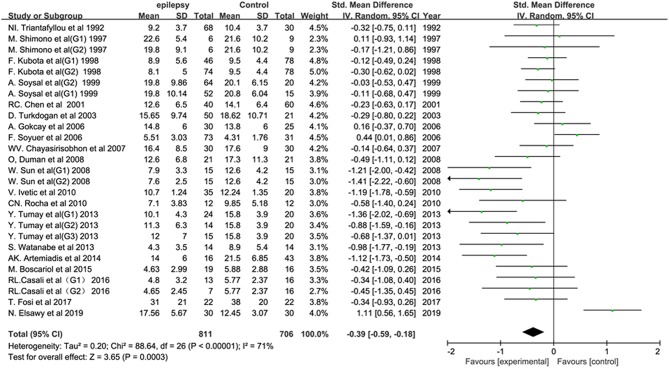
Forest plot of the overall P300 amplitude in epilepsy-related studies. The square size reflects the study's weight. Each horizontal line represents the 95% confidence interval of the standardized mean difference. A diamond shape represents the pooled standardized mean difference. SMD, standardized mean difference; CI, confidence interval.

Twenty-six studies reported the P300 latency in epileptic patients. The pooled results showed a statistically significant difference in the P300 latency between epileptic patients and healthy controls (SMD: 0.78; 95% CI: 0.57, 0.99; *I*^2^ = 83%). In other words, individuals with epilepsy tended to have a longer P300 latency than controls. Twenty studies reported the P300 amplitude in epileptic patients. The combined analysis showed a lower P300 amplitude in individuals with epilepsy than in controls (SMD: −0.39; 95% CI: −0.59, −0.18; *I*^2^ = 71%) (Figure 2). The heterogeneity was significant for both the latency and amplitude of the P300.

### Subgroup Analysis Based on Age Group

Of the studies reporting the P300 latency, 10 studies were conducted in children and adolescents, and 16 studies focused on adults. Both children (SMD: 0.54; 95% CI: 0.42, 0.67; *I*^2^ = 19%) and adults (SMD: 1.00; 95% CI: 0.64, 1.37; *I*^2^ = 90%) with epilepsy had a longer P300 latency than controls in our meta-analysis. Of the studies reporting the P300 amplitude, six studies were carried out in children, and 14 were in adults. Compared with healthy controls, a lower P300 amplitude was observed in both children (SMD: −0.27; 95% CI: −0.51, −0.04; *I*^2^ = 0) and adults (SMD: −0.43; 95% CI: −0.69, −0.16; *I*^2^ = 78%) with epilepsy.

### Subgroup Analysis Based on Type of Epilepsy

Subgroup analyses based on the type of seizure were also conducted with the aim of exploring the change in the P300 latency across different epileptic types compared with that in controls. The types of epileptic seizure mainly included temporal lobe epilepsy, idiopathic epilepsy, symptomatic epilepsy, generalized epilepsy and partial epilepsy. Compared with normal controls, a significantly longer P300 latency was observed in the temporal lobe epilepsy group (SMD: 0.86; 95% CI: 0.25, 1.47; *I*^2^ = 78%; 6 studies), idiopathic epilepsy group (SMD: 1.37; 95% CI: 0.46, 2.27; *I*^2^ = 93%; 7 studies), symptomatic epilepsy group (SMD: 1.46; 95% CI: 0.71, 2.21; *I*^2^ = 90%; 4 studies), generalized epilepsy group (SMD: 1.52; 95% CI: 0.46, 2.58; *I*^2^ = 95%; 7 studies) and partial epilepsy group (SMD: 0.87; 95% CI: 0.39, 1.35; *I*^2^ = 87%; 7 studies). The heterogeneity was still significant in all types of epileptic seizures after subgroup analysis.

### Sensitivity Analysis

We carried out sensitivity analysis by removing one study at a time with the aim of assessing the influence of each included study on the overall results. The significantly longer P300 latency and lower P300 amplitude seen in the epilepsy group never changed throughout our sensitivity analysis. Therefore, the results of the present meta-analysis were reliable and stable.

### Publication Bias

**Figure 3:**
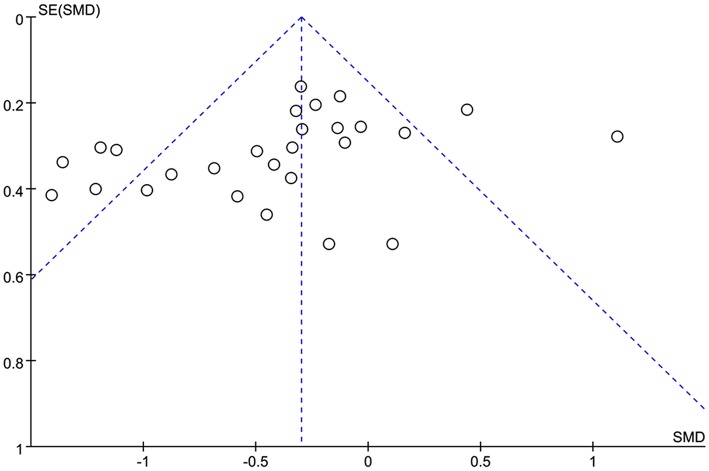
Funnel plot of the selected studies. This analysis suggests no obvious publication bias. SMD, standardized mean difference.

A funnel plot showing the association between the P300 latency and the P300 amplitude (Figure 3) and epilepsy were drawn. In general, it seems that no obvious publication bias was observed in our meta-analysis.

## Discussion

The main results of the present meta-analysis indicated that epileptic patients have a longer P300 latency and a lower P300 amplitude than controls. Subgroup analysis based on age group demonstrated that these differences can also be observed in both children and adult patients compared with healthy controls. In addition, the P300 latency was longer in patients with the five main types of epileptic seizures than in controls.

The P300 is one of the most important ERP components that is used to evaluate cognitive function, such as attention, working memory, and concentration (12, 45). The P300 component has been considered a potential marker of cognitive dysfunction, and it is mostly elicited by the oddball paradigm and appears approximately 300 ms after an infrequent stimulus. The waveform of the P300 component is described by its amplitude and latency. The P300 amplitude represents the degree of information processing, which is also associated with the amount of attentional resources allocated to a task and the level of superior cognitive function (46). Previous studies have demonstrated that the process of selective attention, stimulus evaluation time and working memory updating may be associated with the P300 latency. The prolongation of the P300 latency reflects bad cognitive performance (47, 48). The prevalence of cognitive impairment in epilepsy is estimated to reach 70–80% (3). The early diagnosis and intervention of cognitive impairment in epilepsy is essential for patients to improve their quality of life. Currently, psychological test scales are the main tools for evaluating cognitive dysfunction in epileptic patients. Over the past three decades, many studies have focused on the application of the P300 in epilepsy, with the aim of evaluating neurocognitive function in patients; however, the results have been inconsistent and even contradictory.

A previous investigation showed that brain damage in regions important for cognitive development in epileptic children may lead to neural network impairment and extratemporal abnormalities, which are associated with language impairment and deficits in auditory processing (49). Such abnormalities may have a major influence on cognitive processes and behavior (50). In P300-related studies in epileptic children, Naganuma et al. found that epileptic children had a significantly longer P300 latency than controls (43). Similar conclusions were replicated by subsequent studies (34, 51). However, in several other previous studies, no differences were observed between the epilepsy group and controls in terms of the cognitive potential P300 (22, 30, 52). The results of P300-related studies based on epileptic adults were also controversial and conflicting. Additionally, the latency and amplitude of the P300 in epileptic patients has been identified as being associated with the type of seizure. However, there were inconsistent results across studies. Takhirovna et al. found that the P300 latency in patients with generalized seizures was significantly longer than that in patients with partial seizures (21). Kubota et al. found that there were no significant differences in terms of the P300 latency and P300 amplitude between either the unmedicated partial seizure group or the unmedicated generalized seizure group and the control group (38). The sample size of some studies was too small to have enough statistical power, which may account for the different results. Thus, a total of 27 studies containing 1,513 epileptic patients and 1,124 healthy controls were involved in the present meta-analysis, which can overcome this limitation. The results of our study confirmed the significant role of the P300 latency and P300 amplitude in the cognitive assessment of epileptic patients. Compared with controls, a prolonged P300 latency and a reduced P300 amplitude were found in epileptic patients, both in children and adults. We also found that longer P300 latency and lower P300 amplitude were more obvious in epileptic adults than in children. Thus, we hypothesize that cognitive impairment in adults with epilepsy is more severe than that in epileptic children. In addition, the latency and amplitude of the P300 may be associated with the type of epileptic seizure. Casali et al. found that children with temporal lobe epilepsy (TLE) had a significantly longer P300 latency than controls; however, children with benign childhood epilepsy with centrotemporal spikes (BECTS) did not have a longer P300 latency (13). The latency and amplitude of the P300 may vary largely due to patients having different types of epileptic seizures. Thus, subgroup analyses based on epileptic seizure type were carried out. We found that the P300 latency was significantly longer in patients with all types of epilepsy than in controls. In the present meta-analysis, the prolongation of the P300 latency was most obvious in the generalized epilepsy group, indicating that the cognitive impairment was most severe in generalized epilepsy. Additionally, recent progress revealed the effects of AEDs on cortical activity and connectivity which could be measured by EEG (53). This finding may partly explain the prolongation of p300 latency and reduction of p300 amplitude in epileptic patients due to AEDs treatment.

The heterogeneity was significant in the present meta-analysis. We did not find the potential source of heterogeneity by subgroup analysis or sensitivity analysis. Previous studies have found that the waveform of the P300 can be easily influenced by various factors, such as the complexity of the target stimulus, type of response required, modality, drugs and IQ (54, 55). The location of the electrodes, stimulus frequency and stimulus intensity were widely variable across studies, which may also contribute to the heterogeneity. Recent progress has indicated that the P300 may be a attractive approach to assess cognitive impairment in epilepsy. Our meta-analysis further confirmed this hypothesis. However, there is still a long way to go. The paradigm, target stimulus, location of the electrodes, stimulus frequency and stimulus intensity should be unified first before the P300 can be applied in the cognitive assessment of epilepsy.

Several limitations should be acknowledged in the explanation of our results. First, a meta-analysis may be biased when the literature search fails to identify all relevant studies. However, access to unpublished articles remains difficult, which might be a potential limitation of our study. Second, subgroup analysis based on drug type, disease duration and treatment course was not performed due to the limited studies. Third, the heterogeneity was persistently significant in our study.

## Conclusion

In conclusion, this work revealed that the P300 latency was significantly longer and the P300 amplitude was significantly lower in the epileptic group than in the controls, both in adults and children. This prolongation of the P300 latency can be observed across different types of seizures, such as temporal lobe epilepsy, idiopathic epilepsy, symptomatic epilepsy, generalized epilepsy and partial epilepsy. This study revealed that epileptic patients have abnormalities in the P300 component of event-related potentials, which may reflect deficits in cognitive function. Thus, the P300 may be a potential objective approach for evaluating cognitive function in epileptic patients. Note that more well-designed studies with a large sample size are requested to test our findings.

## Author Contributions

RZ, QC, and WL designed the study, reviewed the literature, conducted the statistical analysis, and drafted of the manuscript collectively. JL, GL, and RZ performed summary tables, edited pictures, and discussed on the manuscript. ML and RZ contributed significantly to the revision of the final manuscript.

### Conflict of Interest Statement

The authors declare that the research was conducted in the absence of any commercial or financial relationships that could be construed as a potential conflict of interest.
